# Effects of light fractionation and different fluence rates on photodynamic therapy with 5-aminolaevulinic acid *in vivo*

**DOI:** 10.1038/sj.bjc.6600910

**Published:** 2003-04-29

**Authors:** P Babilas, V Schacht, G Liebsch, O S Wolfbeis, M Landthaler, R-M Szeimies, C Abels

**Affiliations:** 1 Department of Dermatology, University of Regensburg, Franz-Josef-Strauss-Allee 11, 93042 Regensburg, Germany; 2 Institute of Analytical Chemistry, Chemo- and Biosensors, University of Regensburg, Franz-Josef-Strauss-Allee 11, 93042 Regensburg, Germany

**Keywords:** amelanotic melanoma, protoporphyrin IX, oxygen, microcirculation, tumour

## Abstract

To improve efficacy of photodynamic therapy (PDT) with intravenously administered 5-aminolaevulinic acid (ALA) fractionating the light dose or reducing the light intensity may be a possibility. Therefore, Syrian Golden hamsters were fitted with dorsal skinfold chambers containing an amelanotic melanoma (*n*=26). PDT was performed (100 mW cm^−2^, 100 J cm^−2^, continuously or fractionated, and 25 mW cm^−2^, 100 J cm^−2^; continuously or fractionated) using an incoherent light source following i.v. application of ALA. Following fractionated irradiation, the light was paused after 20 J cm^−2^ for 15 min. Prior to and up to 24 h after PDT tissue, *p*O_2_ was measured using luminescence lifetime imaging. The efficacy was evaluated by measuring the tumour volume of amelanotic melanoma cells grown subcutaneously in the back of Syrian Golden hamsters (*n*=36). Only high-dose PDT resulted in a significant decrease of *p*O_2_. Irrespective of the mode of irradiation only high-dose PDT induced complete remission of all tumours (13 out of 13). It could be shown that low-dose PDT failed to induce a significant decrease of *p*O_2_. No significant effect of fractionated irradiation was shown regarding the therapeutic efficacy 28 days after PDT. Thus performing a fractionated PDT with ALA or reducing the light intensity seems not to be successful in clinical PDT according to the present data.

Photodynamic therapy (PDT) involves the generation of reactive oxygen species after activation of a photosensitiser by light (Pass, 1993; van Hillegersberg *et al*, 1994; Dougherty *et al*, 1998). Since the only clinically approved systemic photosensitiser, Photofrin®, causes generalised cutaneous photosensitivity up to 4–6 weeks following PDT, 5-aminolaevulinic acid (ALA) has raised hope as a promising photosensitiser for PDT (Peng *et al*, 1997). The endogenous photosensitiser ALA is a haem precursor and induces the synthesis of porphyrins (protoporphyrin IX, PpIX) in mitochondria-containing cells (Williams, 1990). A selectivity of ALA-induced porphyrins is described in neoplastic tissue (Abels *et al*, 1994; Ackermann *et al*, 1998; Langer *et al*, 1999) as well as in malignant cells (Rittenhouse-Diakun *et al*, 1995) and provides the basis for clinical use of ALA-based photodynamic therapy (Mlkvy *et al*, 1995) and diagnosis (Kriegmair *et al*, 1995). ALA is water soluble and can therefore be administered either systemically (Grant *et al*, 1993) or topically (Szeimies *et al*, 1994).

However, PDT with intravenously administered ALA failed to be as successful as PDT with Photofrin® in an amelanotic melanoma (Abels *et al*, 1997a). To improve efficacy of ALA–PDT fractionating the light dose or reducing the light intensity might be a possibility (Gibson *et al*, 1990; Messmann *et al*, 1995; de Bruijn *et al*, 1999; Iinuma *et al*, 1999). Splitting the light dose into fractions by pausing the light source for a certain time is supposed to enable reoxygenation of the irradiated tissue and may increase the generation of singlet oxygen thus enhancing PDT (Hua *et al*, 1995; Robinson *et al*, 2000; Pogue *et al*, 2001). In addition, new PpIX might be formed during the irradiation break further contributing to the generation of reactive oxygen species during the second irradiation turn (de Bruijn *et al*, 1999; Robinson *et al*, 2000). A low-dose PDT is supposed to result in a slower oxygen consumption and therefore allows oxygen to be continuously supplied compared with high-dose PDT (Hua *et al*, 1995; Robinson *et al*, 2000; Pogue *et al*, 2001). Therefore, the oxygen concentration, for example, partial oxygen pressure (*p*O_2_), in the tissue is critical and the measurement of tissue oxygenation is of great importance. It is well known that the *p*O_2_ in normal tissue is different from the *p*O_2_ in tumour tissue. While in normal tissue, the median *p*O_2_-values range from 24 to 66 mmHg, the value in the respective malignant tissue is ⩽20 mmHg (Vaupel *et al*, 1989).

In this study, the *p*O_2_ in tumour and surrounding tissue was measured to evaluate the therapeutic effect of different dosimetric protocols for ALA–PDT *in vivo*. The technique of measuring the tissue *p*O_2_ by planar optical sensors and luminescence lifetime imaging (Liebsch *et al*, 2000, 2001) allows a spatially highly resolved mapping of two-dimensional *p*O_2_-distribution within the chamber tissue in a noninvasive way. In addition, the *p*O_2_-measurements were then correlated with tumour growth after PDT with the respective dosimetric protocol.

## MATERIALS AND METHODS

### Animal and tumour model

For experiments male Syrian Golden hamsters of 30–40 g body weight (b.w.) (Breeder: Fa. Harlan-Winkelmann GmbH, Borchen, Germany) and cells of the amelanotic hamster melanoma (A-Mel-3) were used (Fortner *et al*, 1961). The animals were housed in single cages and had free access to food and water.

For the *p*O_2_-measurements, the animals were fitted with titanium dorsal skinfold chambers obtained from the Institute for Surgical Research, Munich, Germany (*n*=26). They tolerated the chambers well and showed no signs of discomfort. Amelanotic melanoma cells (A-Mel-3) (Fortner *et al*, 1961) were implanted (approximately 2 × 10^5^ A-Mel-3 cells in suspension) in the dorsal skinfold chamber 48 h after surgical preparation of the chambers exhibiting an intact microcirculation (for details see, Endrich *et al*, 1980). At 5 days after tumour cell implantation, permanent indwelling catheters (PE-10, 0.4 mm i.d., Portex, Hythe, UK) were inserted into the right carotid artery and right external jugular vein under ketamine/xylazine anesthesia (100 and 8 mg kg^−1^ b.w. i.p.).

To evaluate the impact of the different irradiation protocols on tumour growth 5 × 10^6^ amelanotic melanoma cells were implanted subcutaneously in the shaved and chemically depilated (Pilca med, Olivin, Hamburg, Germany) dorsal skin of the hamsters (*n*=36) under ketamine/xylazine anaesthesia (100 and 8 mg kg^−1^ b.w. i.p.) (Dellian *et al*, 1995; Abels *et al*, 1997a, 1997b). The take rate of the tumours following implantation was 100%. After 4 days of growth, the tumours had grown to a mean volume of approximately 100 mm^3^ (mean tumour volume, 100±7.5 mm^3^). Permanent indwelling catheters (PE-10, 0.4 mm i.d., Portex, Hythe, UK) were inserted into right external jugular vein under ketamine/xylazine anaesthesia (100 and 8 mg kg^−1^ b.w. i.p.) and removed after ALA application.

All experiments have been carried out with ethical committee approval and meet the standards required by the UKCCCR guidelines (Workman *et al*, 1988). In addition, each procedure was approved by the regional authorities according to the German animal care regulations, which are in accordance with the international guidelines of animal care and use in scientific experiments (AZ 621 2531.1-28/00).

### Preparation and administration of ALA

5-Aminolaevulinic acid as a hydrochloride salt (MW 168) was obtained from Merck (Darmstadt, Germany), dissolved in NaCl (pH 6.5) at a concentration of 100 mg ml^−1^ and used immediately. 5-Aminolaevulinic acid was administered intravenously in a dose of 500 mg kg^−1^ b.w. according to previous experiments (Abels *et al*, 1994, 1997a; Szeimies *et al*, 1995). The conscious animals did not show any signs of discomfort during injection of the ALA-solution as previously reported by Edwards *et al* (1984).

### *p*O_2_-Measurement

The *p*O_2_-measurement was performed using luminescence lifetime imaging of a luminescent oxygen indicator, which is immobilised in an oxygen permeable sensor foil (for details see, Liebsch *et al*, 2000, 2001). The indicator is capable of translating the respective oxygen partial pressure into a light signal. The optical *p*O_2_-sensor foil (platinum(II)-octaethyl-porphyrin in polystyrene) was calibrated *in vitro* and attached to the cover glass of the chamber preparation in direct contact with the surface of the chamber tissue. Thus, the *p*O_2_ of the tissue surface can be measured at any time noninvasively. PDT can be performed through the transparent sensor foil because irradiation does not damage the luminescent oxygen sensor (data not shown). A transillumination image can be generated and overlaid with the respective *p*O_2_-image.

The hamsters were fixed in a Perspex tube placed on a motorised, computer-controlled stage (Spindler & Hoyer GmbH, Göttingen, Germany). The temperature of the dorsal skinfold chamber was kept constant by a custom-made water bath at 29°C. For intravital microscopy (IVM), a Zeiss Axiotech Vario 100 HD microscope was modified for time-resolved imaging of luminescent sensor foils. A fast gateable CCD-camera (SensiMod, PCO, Kehlheim, Germany) and a pulsed light source were mounted to the microscope. Green excitation light (535 nm) was reflected by a dichroic mirror (Deep Orange, O590, Balzers) to the *p*O_2_-sensor foil of the observation chamber of the dorsal skinfold chamber. The sensor luminescence was optically filtered with a long-pass filter (RG630, Fa. Schott, Mainz, Germany) and detected via rapid lifetime determination (RLD) (for details see, Liebsch *et al*, 2000, 2001).

Prior to PDT, a transillumination image and a *p*O_2_-image were generated. At 15 min, 1, 2 and 24 h after PDT and, in case of fractionated irradiation, in the irradiation break *p*O_2_-measurements were performed.

### Photodynamic therapy

For light irradiation the animals wearing a transparent dorsal skinfold chamber were immobilised in a Perspex tube. The animals were randomly assigned to the different groups according to the different protocols: control (I); high-dose PDT (100 mW cm^−2^, 100 J cm^−2^), continuous irradiation (II); high-dose PDT (100 mW cm^−2^, 100 J cm^−2^), fractionated irradiation (III); low-dose PDT (25 mW cm^−2^, 100 J cm^−2^), continuous irradiation (IV); low-dose PDT (25 mW cm^−2^, 100 J cm^−2^), fractionated irradiation (V). At the time of highest fluorescence intensity, 150 min after intravenous ALA application (500 mg kg^−1^ b.w), the irradiation was performed with an incoherent light source (*λ*_em_=580–740 nm, PDT 1200, Waldmann, Germany) as published previously (Abels *et al*, 1994, 1997a; Szeimies *et al*, 1995). The light dose of 100 J cm^–2^ was kept constant in all groups. Following the fractionated irradiation, the light was paused after a light dose of 20 J cm^–2^ for a break of 15 min before the remaining 80 J cm^–2^ was applied. The total duration of PDT light treatment was 16.6 min using high-PDT-dose rates (100 mW cm^−2^; 100 J cm^−2^), respectively and 66.6 min using low-PDT-dose rates (25 mW cm^−2^; 100 J cm^−2^).

The animals bearing a solid subcutaneous tumour were placed in a Perspex tube for light treatment. The distance between the animal body and the tumour was increased by raising a skin flap bearing the tumour through a slit in the Perspex tube and fixing it along a plastic arch with three sutures. At 150 min after intravenous ALA application, irradiation was performed with the incoherent light source. The animals were again assigned to the different groups according to the protocol. In addition, a group with continuous irradiation (25 mW cm^−2^, 100 J cm^−2^) but without ALA and a control group were performed. The tumour volume was measured throughout the complete observation period (28 days). The longer (*l*) and the shorter (*w*) perpendicular axes and the height (*h*) of each tumour were measured with callipers prior to and after PDT at 2–3-day intervals over 28 days. Individual tumour volume was calculated using the formula *Clwh*, where *C* is an empirically determined correction factor of 0.873 (Tomayko and Reynolds, 1989; Weiss *et al*, 1990). Tumour response to PDT was classified as follows: complete remission (disappearance of all signs of tumour), partial remission (more than 50% decrease of the product of the two largest perpendicular tumour diameters for a minimum of 7 days), no changes (less than 50% decrease or less than 25% increase of the product of the two largest perpendicular tumour diameters for a minimum of 5 days or progression (more than 25% increase of the product of the two largest perpendicular tumour diameters) (Livingston and Carter, 1982).

### Statistics

For the graphics and statistical analysis of the data, ORIGIN® (Microcal, Northampton, MA, USA) or SigmaStat® was used. Results are expressed as the mean±standard error of the mean if not indicated otherwise. Differences were considered as significant if *P*<0.05.

## RESULTS

### *p*O_2_ measurements

Figure 1

**Figure 1:**
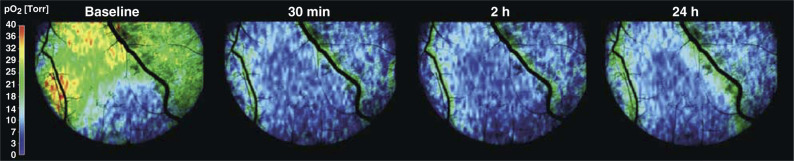
Oxygen maps of the dorsal skinfold chamber with A-Mel-3 tumour prior to and after PDT (100 mW cm^−2^, 100 J cm^−2^, continuous irradiation) over the time (prior to, 30 min, 2 h and 24 h after PDT). The maps are pseudocolour images, the colour bar gives information regarding the colour–*p*O_2_ relation. Prior to PDT, the tumour region can be clearly differentiated from normal tissue because of its blue colour due to the lower oxygen tension. The *p*O_2_ is reduced after irradiation in tumour and surrounding tissue.

shows oxygen maps of the dorsal skinfold chamber with an amelanotic melanoma prior to and after PDT (100 mW cm^−2^, 100 J cm^−2^, continuous irradiation) over the time (prior to, 30 min, 2 and 24 h after PDT). The maps are pseudocolour images, the respective bar is inserted. Prior to PDT, the tumour region can be clearly differentiated from normal tissue because of the lower *p*O_2_ giving in blue colours. The *p*O_2_ is reduced after irradiation in tumour and surrounding tissue.

### High-dose PDT

Figure 2

**Figure 2:**
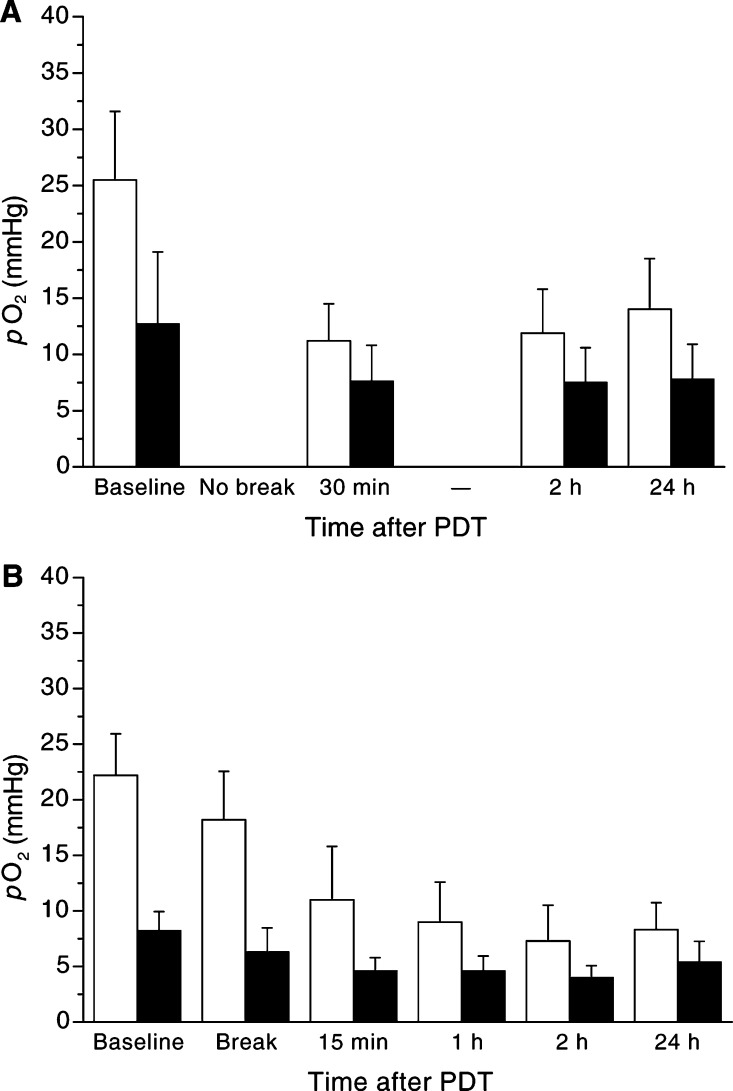
*p*O_2_ was measured prior to and 15 min, 1 h (30 min), 2 h and 24 h (if fractionated additionally in the light pause) after high-dose PDT (100 mW cm^−2^, 100 J cm^−2^) in tumour tissue (▪) and in surrounding tissue (□) after continuous (**A**, *n*=7) or fractionated (**B**, *n*=6) irradiation. A decrease of *p*O_2_ in tumour and surrounding tissue is shown following high-dose PDT (median±s.e.m.).

shows the *p*O_2_-measurements following high-dose PDT (100 mW cm^−2^, 100 J cm^−2^) irradiated either continuously (Figure 2A) or fractionated (Figure 2B).

Continuous irradiation induced a significant decrease of *p*O_2_ at any time in surrounding tissue and in tumour tissue: following PDT, *p*O_2_ decreased from 25.5±6.1 mmHg (baseline) to 11.2±3.3 mmHg (30 min) followed by a slight increase to 14±4.5 mmHg (24 h) in surrounding tissue. In tumour tissue, *p*O_2_ decreased from 12.7±6.4 mmHg (baseline) to 7.6±3.2 mmHg (30 min) to remain at this level up to 24 h (Figure 2A).

The fractionated irradiation induced a decrease of *p*O_2_ in surrounding tissue and in tumour tissue as well (Figure 2B). Following PDT, *p*O_2_ decreased from 22.2±3.7 mmHg (baseline) to 18.2±4.3 mmHg (in the irradiation break), to 11.0±4.8 mmHg (15 min) and to 7.3±3.2 mmHg (2 h) followed by a slight increase to 8.3±2.4 mmHg (24 h) in surrounding tissue. In tumour tissue, *p*O_2_ decreased from 8.2±1.7 mmHg (baseline) to 6.3±2.2 mmHg (in the irradiation break), and to 4.0±1.1 mmHg (2 h) followed by a slight increase to 5.4±1.8 mmHg (24 h) (Figure 2B).

In surrounding tissue, there was a difference detectable following high-dose PDT with continuous irradiation as compared to high-dose PDT with fractionated irradiation. The *p*O_2_ recovered 24 h following irradiation to 55% of the baseline-value in surrounding tissue following continuous irradiation, whereas following fractionated irradiation, the *p*O_2_ showed just a slight increase to 37% of the baseline-value 24 h following irradiation (Figure 2). In tumour tissue *p*O_2_ showed no significant difference following continuous or fractionated irradiation as the *p*O_2_ recovered 24 h following irradiation to 61% and to 66%, respectively, of the baseline-value (Figure 2).

### Low-dose PDT

Figure 3

**Figure 3:**
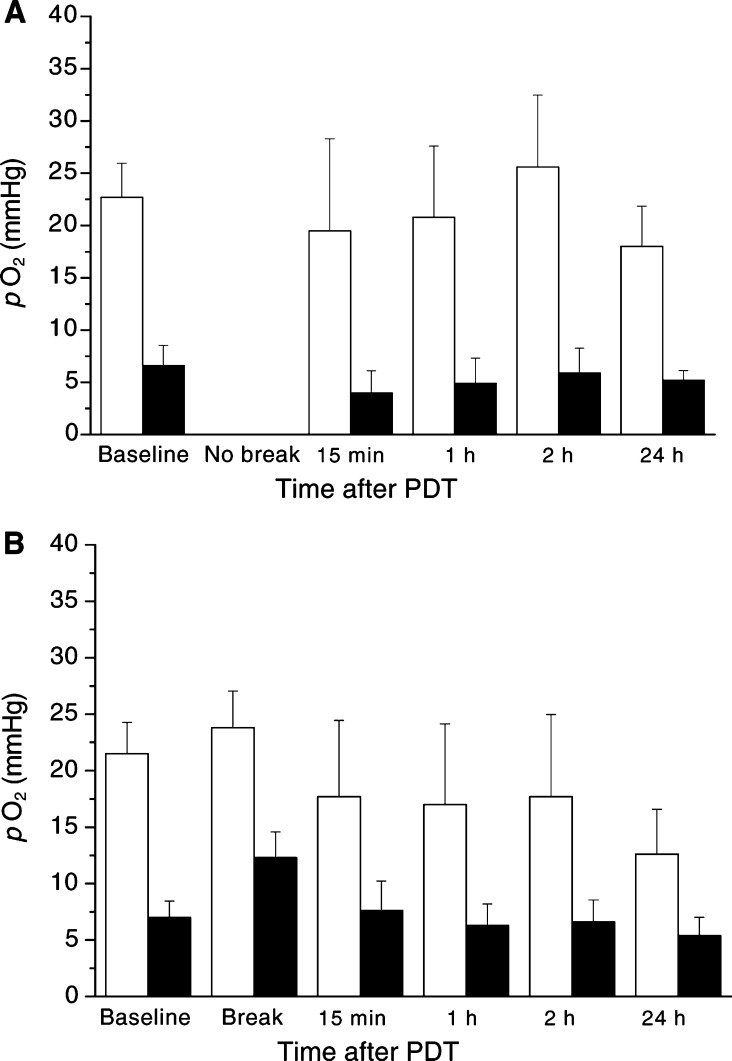
*p*O_2_ was measured prior to and 15 min, 1 h, 2 h and 24 h (if fractionated additionally in the light pause) after low-dose PDT (25 mW cm^−2^, 100 J cm^−2^) in tumour tissue (▪) and in surrounding tissue (□) after continuous (**A**, *n*=7) or fractionated (**B**, *n*=6) irradiation (median±s.e.m.).

shows the *p*O_2_-measurements following low-dose PDT (25 mW cm^−2^, 100 J cm^−2^) irradiated either continuously (Figure 3A) or fractionated (Figure 3B).

Continuous irradiation during PDT induced a slight decrease of *p*O_2_ in surrounding tissue and in tumour tissue: following PDT, *p*O_2_ decreased from 22.7±3.3 mmHg (baseline) to 19.5±8.8 mmHg (15 min), followed by a slight increase to 20.8±6.8 mmHg (1 h) and to 25.6±6.8 mmHg (2 h), followed by a decrease to 18±3.8 mmHg (24 h) in surrounding tissue. The slight increase of tissue *p*O_2_ in surrounding tissue 2 h after treatment is because of a hyperaemic reaction. In tumour tissue, *p*O_2_ decreased from 6.6±1.9 mmHg (baseline) to 4.0±2.0 mmHg (15 min), followed by an increase after 2 h, followed by a decrease to 5.2±0.9 mmHg (24 h) (Figure 3A).

The fractionated irradiation induced a slight decrease of *p*O_2_ in surrounding tissue and in tumour tissue as well (Figure 3B). Following PDT, *p*O_2_ increased from 21.5±2.7 mmHg (baseline) to 23.8±3.2 mmHg (in the irradiation break), decreased to 17.7±6.7 mmHg (15 min/2 h) followed by a slight decrease after 24 h in surrounding tissue. In tumour tissue, *p*O_2_ increased from 7.0±1.5 mmHg (baseline) to 12.3±2.2 mmHg (in the irradiation break), followed by a decrease to 7.6±2.6 mmHg (15 min) and to 6.3±1.9 mmHg (1 h), followed by a slight increase to 6.6±1.9 mmHg (2 h), followed by a slight decrease to 5.4±1.6 mmHg (24 h) (Figure 3B).

Remarkable is the fact that the *p*O_2_ in the irradiation break increased in tumour and surrounding tissue following fractionated low-dose PDT. On the contrary, following high-dose PDT, the *p*O_2_ decreased in the irradiation break in tumour and surrounding tissue (Figure 2B and Figure 3B).

### Tumour growth curves

Figure 4

**Figure 4:**
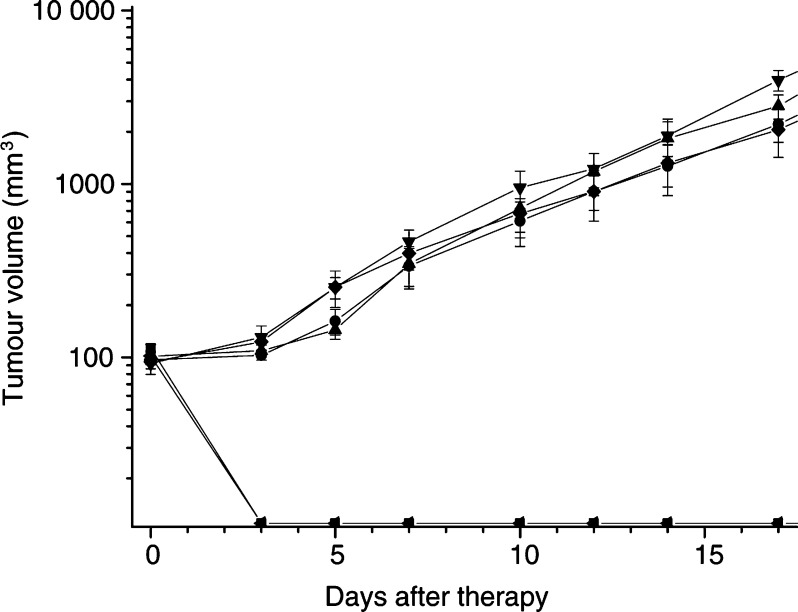
A-Mel-3 amelanotic melanomas were implanted subcutaneously in the dorsal skinfold of Syrian Golden hamsters (*n*=38). At 150 min after i.v. ALA application (500 mg kg^−1^ b.w.) irradiation was performed. Six groups were formed according to the protocols: (♦) control (I, *n*=6); (◂) high-dose PDT (100 mW cm^−2^, 100 J cm^−2^), continuous irradiation (II, *n*=7); (▪) high-dose PDT (100 mW cm^−2^, 100 J cm^−2^), fractionated irradiation (III, *n*=6); (•) low-dose PDT (25 mW cm^−2^, 100 J cm^−2^), continuous irradiation (IV, *n*=7); (▴) low-dose PDT (25 mW cm^−2^, 100 J cm^−2^), fractionated irradiation (V, *n*=6); (▾) low-dose PDT (25 mW cm^−2^, 100 J cm^−2^), continuous irradiation without ALA (VI, *n*=6)]. Tumour volumes were recorded throughout the complete observation period (28 days) (median±s.e.m.).

shows the tumour growth curves of the different experimental groups. Prior to PDT, there was no statistical difference of the tumour volume of any of the experimental groups including the control group (I–VI) (Figure 4). Prior to high-dose PDT (100 mW cm^−2^, 100 J cm^−2^, groups II and III), the mean volume of the subcutaneously grown tumours was 101±16 mm^3^ in group II and 113±6 mm^3^ in group III. Group II, treated with continuous high-dose PDT, as well as group III, treated with fractionated high-dose PDT, showed no signs of tumour 3 days after therapy, instead a massive scar formation developed. There was no recurrence up to the end of the observation period. Thus, high-dose PDT induced complete remission of all tumours (13 out of 13) (Table 1

**Table 1:** Complete remission of all tumours induced by high-dose PDT

**Groups**	**ALA** (**mg kg^−1^ b.w.**)	**Light intensity** (**mW cm^−2^**)	**Irradiation**	**Complete remission**	**Partial remission**	**No change**	**Progression**
I	—	—	—	0/6	0/6	0/6	6/6
II	500	100	Continuous	7/7	0/7	0/7	0/7
III	500	100	Fractionated	6/6	0/6	0/6	0/6
IV	500	25	Continuous	0/7	0/7	0/7	7/7
V	500	25	Fractionated	0/6	0/6	0/6	6/6
VI	—	25	Continuous	0/6	0/6	0/6	6/6

PDT=photodynamic therapy. Data represent the therapeutic outcome after 28 days of a subcutaneously implanted amelanotic malignant melanoma (A-Mel-3) in Syrian Golden hamsters following PDT with 5-ALA i.v. (500 mg kg^−1^ b.w.) and irradiation (fluence: 100 J cm^−2^) 150 min thereafter using incoherent light source and different dosimetric parameters.

). Irradiation either continuous or fractionated did not change the therapeutic outcome in these groups (Figure 4).

Prior to low-dose PDT (25 mW cm^−2^, 100 J cm^−2^, groups IV and V), the mean volume of the subcutaneously grown tumours was 97±5 mm^3^ in group IV and 101±7 mm^3^ in group V. Group IV, treated with continuous low-dose PDT, as well as group V, treated with fractionated low-dose PDT, showed a reduced mean tumour volume at day 3 (group IV: 102±6 mm^3^; group V: 108±8 mm^3^) and day 5 (group IV: 161±27 mm^3^; group V: 143±16 mm^3^) as compared to control group (I) (day 3: 123±13 mm^3^; day 5: 254±60 mm^3^). From day 9 up to the end of the observation period, there was no difference in tumour growth between group IV or group V and the control group (I) detectable (Figure 4). There was no significant difference in tumour growth curves following either continuous or fractionated irradiation (Figure 4). In contrast to high-dose PDT, low-dose PDT resulted in progression of all tumours (13 out of 13), independent of the irradiation protocol (Table 1).

Prior to continuous low-dose PDT without ALA (25 mW cm^−2^, group VI), the mean volume of the subcutaneously grown tumours was 92±12.5 mm^3^. During the whole observation period, there was no significant difference in tumour growth between group VI and the control group (I) (Figure 4). The tumour growth curve showed an exponential trend. Low-dose PDT without ALA resulted in progression of all tumours (six out of six) (Table 1).

The subcutaneously grown tumours of the control group (I) showed a mean tumour volume of 94±3.4 mm^3^ at the beginning of the observation period. The tumour growth curves showed an exponential growth (Figure 4). All tumours showed progression (six out of six) (Table 1).

## DISCUSSION

This study shows that only high-dose PDT (100 mW cm^−2^; 100 J cm^−2^) with ALA results in a significant decrease of *p*O_2_ 24 h after PDT as compared to low-dose PDT (25 mW cm^−2^, 100 J cm^−2^) (Figure 2 and 3). Fractionation of PDT did not show any effect regarding the *p*O_2_ in tumour tissue. However, following fractionated irradiation, the *p*O_2_ in surrounding tissue decreased to a significant lower value 24 h after either high-dose or low-dose PDT as compared to tumour tissue (Figure 2 and 3).

Only high-dose PDT induced complete remission of all tumours (13 out of 13). Neither continuous nor fractionated irradiation did change the therapeutic outcome after high-dose or low-dose PDT (Figure 4).

The dorsal skinfold chamber model was used in the present study because this tumour model is well established to investigate the microcirculatory effects on tumour and normal tissue following different therapeutic approaches (Dellian *et al*, 1995; Schacht *et al*, 2001). In addition, measuring the tumour volume of the subcutaneously implanted amelanotic melanoma after PDT has been studied as well to evaluate the efficacy of different anticancer treatments (Weiss *et al*, 1990; Szeimies *et al*, 1995; Dellian *et al*, 1996; Abels *et al*, 1997a, 1997b).

Photodynamic therapy was performed according to previous studies (Abels *et al*, 1994, 1997a; Szeimies *et al*, 1995). Since in the literature it is reported that low-dose PDT or fractionated irradiation may be more effective as compared to high-dose PDT or continuous irradiation (Gibson *et al*, 1990; Messmann *et al*, 1995; de Bruijn *et al*, 1999; Iinuma *et al*, 1999), PDT dosimetry was performed with a light intensity of either 100 or 25 mW cm^−2^ and light was applied either continuously or fractionated according to the experiments of Curnow *et al* (2000) and de Bruijn *et al* (1999).

The efficacy of ALA–PDT was evaluated by measuring the tissue *p*O_2_ (Blumenroder *et al*, 1997; Hartmann *et al*, 1997; Curnow *et al*, 2000; Niedre *et al*, 2002) since tissue *p*O_2_ correlates with a functional microcirculation (Vaupel, 1994). The technique of measuring the tissue *p*O_2_ by luminescence lifetime imaging using planar oxygen sensors allows a spatially highly resolved and time-resolved mapping of two-dimensional *p*O_2_-distribution within the entire chamber tissue in a noninvasive way (Liebsch *et al*, 2000, 2001).

Thus, two independent parameters to assess PDT efficacy were determined: the tissue *p*O_2_ can be considered as a short-term parameter (up to 24 h after PDT), whereas the tumour growth curves represent the long-term parameter (up to 28 days after PDT).

In Figure 2A and 2B, *p*O_2_ in tumour and surrounding tissue decreases up to 24 h following continuous or fractionated high-dose *PDT*. This result is in agreement with the literature. McIlroy *et al* (1998) described a 10-fold decrease of the oxygen level in normal rat liver following PDT with ALA (100 mW, 60 J) (McIlroy *et al*, 1998). The decline of *p*O_2_ following PDT could be caused by photochemical consumption of molecular oxygen (Busch *et al*, 2000) or by the lack of oxygen supply caused by massive microvascular damage following high-dose PDT (Castellani *et al*, 1963; Vaupel *et al*, 1977, 1981, 1989). The fragile microvasculature of experimental and human solid tumours is characterised already in very early growth stages by structural and functional abnormalities (Vaupel *et al*, 1989). The structural abnormalities can result in deficient endothelial lining or basement membrane, in loss of vessel hierarchy or in heterogeneous vascularisation, whereas the functional abnormalities can lead to an increased vascular fragility, to an increase of viscous resistance with micro- and macrothrombosis or to an increased interstitial fluid pressure (Ribbert, 1904; Vaupel *et al*, 1989). These specific characteristics make the microvasculature of experimental and human solid tumours very vulnerable for any treatment targeting the microvasculature, like PDT.

In contrast to other investigations, fractionation of the light dose (20 J cm^−2^, 15 min, 80 J cm^−2^) did not further decrease *p*O_2_ in tumour tissue in the present study. However, in surrounding tissue a further reduction of *p*O_2_ 24 h after PDT is shown (Figure 2B). Curnow *et al* (2000) described using the polarographic Eppendorf *p*O_2_ histograph system in three animals also a benefit of fractionation (5 J, 150 s, 20 J) of PDT with ALA (200 mg kg^−1^) in the normal rat colon. They measured a greater decrease of *p*O_2_ and a greater area of necrosis following fractionated irradiation. However, they did not perform any measurements in tumour tissue. Despite the fact that the Eppendorf *p*O_2_ histograph is considered to be the gold standard for measuring *p*O_2_ in tissue (Nozue *et al*, 1996), it does not allow a spatially and highly time-resolved measurement of the *p*O_2_-value as compared with the method used in this study. Curnow *et al* were not able to accurately ascertain in which tissue layer the polarographic Eppendorf needle is located during measurements. Moreover, they did perform the measurements only at one site per animal. According to the extensive investigations by Vaupel (Vaupel *et al*, 1977, 1981, 1989) measuring just at one point does not represent the entire tissue (Lord and Paoni, 2001). Besides this, the mechanical pressure exerted by placing the needle in the tissue can easily irritate the membrane and therefore change the diffusion capacity of the membrane. The used technique against the background of measuring the *p*O_2_ just in normal tissue and not in tumour tissue makes this study incomparable to the results of our study as described above. Most probably if the study had focused on tumour tissue they would have obtained similar results.

Using *p*O_2_-electrodes, Pogue *et al* (2001) could show in the rat R3230Ac mammary adenocarcinoma after fractionated PDT with Verteporfin® directly after completion of the irradiation a heterogeneous reduction of tissue *p*O_2_, but not complete anoxia. Unfortunately, measurements in surrounding normal tissue were not taken. Nevertheless, using laser Doppler measurements in the same experiments, a significant reduction of blood flow during the initial phase of irradiation could be shown (Pogue *et al*, 2001).

Measuring the diameter of necrosis in tumour and normal tissue following mTHPC-mediated PDT, Tsutsui *et al* (2002) showed a significant enhancement of necrosis produced only in normal tissue and not in tumour tissue following fractionated irradiation with 100 mW. This finding supports our result showing that if an efficient PDT is performed, a fast breakdown of the microcirculation in tumours occurs preventing an additional benefit of fractionated irradiation in tumour tissue, but not in the surrounding tissue with a less fragile microvascular system, which is still functioning, thus supplying oxygen (Djavaheri-Mergny *et al*, 2001; Lord and Paoni, 2001).

Following low-dose PDT (25 mW cm^−2^, 100 J cm^−2^) and continuous irradiation, a slight but not significant reduction of tissue *p*O_2_ can be observed 24 h after treatment in this model (Figure 3A). Since high-dose PDT with continuous irradiation resulted in 100% complete remission (Figure 4), this dosimetry protocol, not inducing complete remission, was chosen to demonstrate a benefit by using fractionated irradiation. As expected, an increase of tissue *p*O_2_ in surrounding tissue (111% of baseline) and an even more pronounced increase in tumour tissue (176% of baseline) could be measured during the irradiation break (Figure 3B). Nevertheless, 24 h after irradiation there is a higher relative decrease of *p*O_2_ in surrounding tissue (58.6% of baseline) as compared to tumour tissue (77.1% of baseline). The increase of tissue *p*O_2_ in tumour and surrounding tissue during the irradiation break can be explained by a reduced cellular respiration break by functioning oxygen supply. This assumption is supported by the fact that ALA is primarily metabolised to porphyrins in the tumour parenchyma (Fritsch *et al*, 1997). Therefore, direct tumour cell toxicity by PDT with ALA is expected. Further evidence is given by measurements of NADH fluorescence to assess acute cellular PDT-induced damage *in vivo*, showing a significant reduction directly after the first fraction of irradiation using Verteporfin® as photosensitiser (Pogue *et al*, 2001). Comparing continuous *vs* fractionated irradiation for low-dose PDT, no difference can be measured regarding *p*O_2_ 24 h after treatment in tumour tissue, but as for high-dose PDT only in the surrounding tissue, difference can be measured.

The tumour growth curves (Figure 4) show a completely different development comparing high-dose PDT (groups II and III) with low-dose PDT (groups IV and V). There was no difference following high-dose PDT regarding continuous or fractionated irradiation, because continuous irradiation induced disappearance of all signs of tumour 3 days after therapy. Thus, any benefit of fractionated irradiation would have been masked. According to previous results using this model (Szeimies *et al*, 1995; Abels *et al*, 1997), the development of the tumour growth curves after high-dose PDT was unexpected, because in these experiments PDT using the same parameters and continuous irradiation yielded only one complete remission out of six subcutaneously implanted amelanotic melanomas. The only experimental difference is the use of an argon-pumped dye laser tuned to 635 nm for irradiation, while in the present investigation an incoherent light source (*λ*=580–740 nm) was employed. Since significant amounts of additional porphyrins, besides PpIX, are formed in this model following systemic application of ALA (Fritsch *et al*, 1997) absorbing beyond 635 nm; the wavelength range of the incoherent light source covers their absorption spectrum, thus leading to a more pronounced photodynamic effect.

In contrast, low-dose PDT with either continuous or fractionated irradiation shows just a transient and small delay of tumour growth at days 3–7 as compared to the control group (Figure 4). Moreover, at day 5 there might be a nonsignificant difference visible between continuous (162±27 mm^3^) or fractionated irradiation (143±17 mm^3^), showing the influence of the significant reduction of *p*O_2_ in the surrounding tissue after 24 h by fractionated low-dose PDT (Figure 3B).

These results regarding the efficacy of low-dose *vs* high-dose PDT with ALA are supported by the findings of Iinuma *et al* (1999), which could demonstrate a 2-log cell kill and a 3-log cell kill for ALA–PDT with either 30 or 100 mW cm^−2^, respectively, in a rat tumour model *in vivo*.

In contrast, Tsutsui *et al* (2002) found a low-dose PDT to be more effective by measuring the depth of necrosis in tumour tissue following mTHPC-mediated PDT. They documented that depth of necrosis following continuous PDT with a low light intensity (5 mW) is significantly larger as compared to PDT with a high light intensity (100 mW) from 2.5 to 30 J total light energy. However, they did not perform an irradiation with a high light dose of 100 J cm^−2^ as carried out in this study.

A 100% complete remission (13 out of 13) was achieved following high-dose PDT (100 mW cm^−2^, 100 J cm^−2^) (Table 1). Both irradiation schemes, continuous and fractionated, turned out to be exactly equivalent concerning the therapeutic efficacy in this model. In contrast, no complete remission was observed after low-dose PDT and this is also independent of the irradiation scheme. Therefore, these results do not support the hypothesis that lower PDT fluence rates are associated with increased efficiency of tumour response (Sitnik and Henderson, 1998). Surprisingly, in this study no complete remission of the radiation induced fibro sarcoma (RIF) tumour was obtained using Photofrin® (5 mg kg^−1^) and high-dose PDT (150 mW cm^−2^, 100 J cm^−2^), whereas in our model using these parameters and Photofrin® in five out of six tumours a complete remission is achieved (Dellian *et al*, 1996).

In conclusion, measuring tissue *p*O_2_ by means of luminescence lifetime imaging proves to be a helpful tool to evaluate the acute effects of PDT on the microcirculation. Only high-dose PDT with a high light intensity and a high light dose induces complete remission in our model. Moreover, it could be shown that fractionating the light dose enhances the effect of PDT only in surrounding but not in tumour tissue, since oxygen supply in tumour tissue is already maximal because of the characteristic structural and functional abnormalities of the tumour microvasculature. In addition, no significant effect of fractionated irradiation was shown in our tumour model regarding the therapeutic efficacy 28 days after PDT. The *p*O_2_-measurements correlate with the measurements of the tumour volume, only PDT inducing a significant reduction of *p*O_2_ in tumour as well as normal tissue 24 h after PDT resulted in complete remission of tumours 28 days after PDT.

Thus, performing a fractionated PDT with ALA or reducing the light intensity and increasing irradiation time seems not successful in clinical PDT according to the present data.
